# Constructing “Packages” of Evidence-Based Programs to Prevent Youth Violence: Processes and Illustrative Examples From the CDC’s Youth Violence Prevention Centers

**DOI:** 10.1007/s10935-016-0423-x

**Published:** 2016-04-01

**Authors:** Beverly Kingston, Martica Bacallao, Paul Smokowski, Terri Sullivan, Kevin Sutherland

**Affiliations:** Center for the Study and Prevention of Violence, Institute of Behavioral Science, University of Colorado Boulder, 1440 15th Street, Boulder, CO 80302 USA; University of Kansas, Lawrence, KS USA; University of North Carolina Chapel Hill, Chapel Hill, NC USA; Virginia Commonwealth University, Richmond, VA USA

**Keywords:** Violence prevention, Evidence-based programs, Comprehensive approach, Community-academic partnerships

## Abstract

This paper describes the strategic efforts of six National Centers of Excellence in Youth Violence Prevention (YVPC), funded by the U.S. Centers for Disease Control and Prevention, to work in partnership with local communities to create comprehensive evidence-based program packages to prevent youth violence. Key components of a comprehensive evidence-based approach are defined and examples are provided from a variety of community settings (rural and urban) across the nation that illustrate attempts to respond to the unique needs of the communities while maintaining a focus on evidence-based programming and practices. At each YVPC site, the process of selecting prevention and intervention programs addressed the following factors: (1) community capacity, (2) researcher and community roles in selecting programs, (3) use of data in decision-making related to program selection, and (4) reach, resources, and dosage. We describe systemic barriers to these efforts, lessons learned, and opportunities for policy and practice. Although adopting an evidence-based comprehensive approach requires significant upfront resources and investment, it offers great potential for preventing youth violence and promoting the successful development of children, families and communities.

## Background

### Introduction

Preventing and reducing the risk for youth violence remains a significant challenge for communities across the country. National statistics for homicide and violence-related behaviors, school violence, and bullying underscore that youth violence is a significant public health concern in the United States. In 2011, homicide victims included approximately 4500 youth ages 15–24, and homicide was the third leading cause of death among youth ages 15–24 after unintentional injuries and suicide (CDC, 2014). The 2013 Youth Risk Behavior Survey indicated that among high school students, 24.7 % reported being in a physical fight one or more times in the past year, and 8.1 % reported being in a physical fight that occurred on school grounds at least once in the 12 months before being surveyed (Kann, Kinchen, & Shanklin, 2014). Prevalence rates for bullying behaviors are also of concern. Nansel et al. (2001) found 29.9 % of surveyed students in the United States reported involvement in bullying situations. Across the United States, bullying victimization rates range from 10 to 28 % (Eaton et al., 2012; Robers, Kemp, & Truman, 2013; Wang, Iannotti, & Nansel, 2009). In addition, one in 14 youth reported missing school at least 1 day in the past 30 days because they were concerned about safety at school or when traveling to school or back home (Kann et al., 2014).

Negative outcomes of youth violence include not only the risk of serious physical injury or death but also struggles in psychosocial adjustment and academic achievement, which may adversely impact future career possibilities and relationships (Bradshaw, O’Brennan, & McNeely, 2008). A growing body of literature highlights the cumulative risk for harmful outcomes attributed to youths’ exposure to multiple types of victimization and adversity (Dodge, Greenberg, & Malone, 2008). For example, researchers have consistently found that multiple adversities in childhood are associated with an increased risk for psychiatric and behavioral problems in childhood, adolescence, and adulthood compared to non-exposed individuals or to those exposed to fewer types of victimization (Copeland, Keeler, Angold, & Costello, 2007; Felitti et al., 1998; Pynoos, Steinberg, Schreiber, & Brymer, 2006).

While there is agreement within the research community that comprehensive approaches using evidence-based programs to reduce the risk for youth violence are needed (Gottfredson, 2001; Jenson & Fraser, 2011), there are few descriptions of what these types of approaches might look like, not to mention how researchers might partner with communities to identify and implement prevention programs that are well-grounded in theory, have empirical evidence of effectiveness, and meet unique community needs. This paper describes the strategic efforts of six National Centers of Excellence in Youth Violence Prevention (YVPC) to select and integrate comprehensive evidence-based program packages to prevent youth violence in their respective communities. Each community was identified based on its high prevalence rates of youth violence, and all selected communities (5 urban and 1 rural) were in low-income areas across the nation. Sites presented a variety of data on the prevalence of youth violence within the selected communities to justify their inclusion. Another requirement of this CDC YVPC initiative was to include a package of evidence-based programs that were directed both toward universal (e.g., delivered to all youth in a population such as a school) and toward populations at high risk for youth violence perpetration. Each site identified prevention programs based on community needs within these guidelines and collected the types of data and specific measures that were most relevant to document potential changes in youth violence perpetration and associated risk and protective factors driven by these prevention efforts (see Matjasko, Massetti, & Bacon, 2016, the introduction to this issue, for additional information about site selection). Each YVPC site aimed to develop an evidence-based program package that was responsive to the specific needs of the community or communities it served.

### What Is an Evidence-Based Comprehensive Approach?

Comprehensive prevention and intervention strategies that make use of the best scientific findings about effective programs and implementation methods to address multiple aspects of a child’s family, peer, school, and community life from early childhood through adolescence will likely have the greatest impact on youth violence at the greatest savings in cost (Coalition for Evidence Based Policy, 2014; Washington State Institute for Public Policy, 2014). For the YVPCs, evidence of effectiveness was defined as at least one publication in a peer-reviewed journal using randomized or rigorous quasi-experimental designs with matched control groups. National recommendations for addressing youth violence and other problem behaviors advocate using a coordinated, comprehensive approach to reduce risk factors and to enhance protective factors at the individual, family, peer, school and community levels (Ridgeway, 2014). Research shows that many of the same risk factors are associated with a wide range of adolescent problem behaviors (e.g., substance abuse, delinquency, teen pregnancy, school dropout, violence, and depression and anxiety) suggesting that targeted risk reduction can affect a broad set of outcomes simultaneously (Herrenkohl, Aisenberg, Williams, & Jenson, 2011).

A comprehensive approach includes complementary components that are designed to work at multiple levels of the social context (e.g., individual, family, peer, school, community) to address the risk and protective factors that impact violence and other problem behaviors. Some of the strongest risk factors predicting violence and other problem behaviors include early and persistent problem behavior (e.g., early involvement in serious offenses and substance use by children under age 12), deviant peer relationships, and parental influences such as lack of parental warmth and inconsistent parental monitoring (Dodge et al., 2008; U.S. Department of Health and Human Services, 2001). Since the levels of these risk and protective factors vary by community, it is important that communities use a data-driven process to understand and prioritize their unique needs (Hawkins et al., 2009) Examination of community needs may also entail identifying and building upon evidence-based prevention programs that are already in place and being implemented with fidelity.

Additionally, designing a comprehensive approach involves ensuring that adequate exposure to the prevention components is provided to a large enough number of people to have the level of saturation necessary to achieve the desired preventive effects. By including program components that are provided universally (e.g., delivered to all youth, regardless of risk) as well as components that are focused on subgroups of youth or families at elevated risk, the likelihood of community-wide reductions in youth violence and other problem behaviors is increased. For example, universal interventions can create a strong foundation for early and more intensive interventions to succeed, while intensive interventions can reduce peer contagion influences that may undermine the impact of universal and early interventions (Osher, Dwyer, & Jimerson, 2006).

Ideally, communities will utilize evidence-based programs and strategies to support their comprehensive approach. There is a rich and growing body of evidence demonstrating that certain programs and practices are effective, both for preventing the onset of problem behaviors and for successfully intervening with youth exhibiting problem behaviors (Greenwood, 2006; Institute of Medicine, 2008; Sherman, Farrington, Welsh, & MacKenzi, 2002). Examples of programs that meet the highest standards of effectiveness can be found on several registries of evidence-based programs (i.e., Blueprints for Healthy Youth Development, 2015a, b; Coalition for Evidence Based Policy, 2015; Office of Justice Programs’ CrimeSolutions.gov [list of effective programs], 2015).

Several models of comprehensive community approaches that advocate using evidence-based programs matched to community needs have been developed and tested (Hawkins et al., 2009; Redmond et al., 2009). These approaches emphasize decision-making by the local community, often in partnership with researchers. For example, Communities That Care is a prevention system that aims to reduce elevated risks, enhance protection, promote healthy youth development and prevent adolescent problem behavior community-wide (Hawkins, Catalano, & Arthur, 2002). It provides a community-level collaborative model for using data to select and implement evidence-based programs based on local needs. The PROSPER partnership model is an evidence-based delivery system for community-based prevention that is designed to decrease adolescent problem behavior in rural and semi-rural communities by utilizing existing systems to deliver evidence-based prevention programs (Spoth, Greenberg, Bierman, & Redmond, 2004).

Developers of these comprehensive systems advocate for integrating community and practitioner perspectives with those of prevention science (Fagan, Hanson, Hawkins, & Arthur, 2009; Spoth & Greenberg, 2011). This requires an understanding of both the barriers and the infrastructures necessary to support practitioners and researchers collaborating to translate science into prevention practice (Saul et al., 2008). Next we provide an overview of some common barriers to adopting an evidence-based comprehensive approach at the community level. This is followed by an overview of key considerations based in implementation science for matching comprehensive packages of evidence-based youth violence prevention and intervention programs with community needs.

### Barriers to an Evidence-Based Comprehensive Approach

Although the federal government has been taking steps to support an evidence-based comprehensive approach, most community-based youth violence prevention efforts fund programs that have not been evaluated, and some communities are still implementing programs that are proven ineffective and even harmful (e.g., Scared Straight and Boot Camps; Elliott, 2013). To illustrate, estimates suggest that evidence-based prevention programs are implemented in only about 10 % of agencies within child public service systems (e.g., child welfare, juvenile justice, mental health) in the United States (Hoagwood & Olin, 2002), and school-based estimates of evidence-based prevention program implementation are even lower (U.S. Department of Education, 2011). For example, the U.S. Department of Education’s evaluation on the use of evidence-based programs in prevention of substance abuse and school crime reported that only 7.8 % of school programs were research based. Of these research-based programs, only 44 % met standards of effective implementation. This low implementation quality is particularly concerning as program fidelity has been linked to positive outcomes (Durlak, 2010; Durlak & DuPre, 2008; Gottfredson & Gottfredson, 2002; Wilson, Lipsey, & Derzon, 2003).

Several overarching systemic barriers affect the successful adoption of an evidence-based comprehensive approach. First, communities and schools often struggle to understand what it means for a program to be defined as “evidence-based.” While the term is now widely used, the actual level of evidence required for certification varies from informal satisfaction surveys, to single studies with non-experimental designs, to multiple randomized control design studies (Elliott, 2013; Tolan, 2014). Adding to this confusion is the fact that there is currently little consensus within the research and practice communities about the scientific standard that should be used to certify an individual program as effective or evidence-based (Elliott, 2013). Depending on the source, the standard varies from any positive effect from any type of study, to consistent positive effects from multiple randomized control trials.

Helping communities to understand that evidence of program effectiveness can be viewed on a continuum can be a useful way to address this confusion and build community capacity to support quality evaluation processes. For example, at the highest end of the continuum of evidence are programs that have been subjected to one or more randomized control trials, with effects sustained for at least 1 year after the program ends, and with replications that show the same positive effects—these programs are experimentally proven. Programs that have some evidence of effectiveness (e.g., single group pre-post test designs) fall towards the lower end of the continuum. These programs provide some evidence of effectiveness but they lack an appropriate comparison group and evidence of a causal effect (Blueprints for Healthy Youth Development, 2015a, b). At the other end of the continuum, there are programs that have strong evidence demonstrating that they are ineffective and even harmful (Puddy & Wilkins, 2011).

Second, communities and schools often lack the resources, capacity and infrastructure to implement an evidence-based comprehensive approach (Catalano et al., 2012). Communities and schools are not empty vessels eagerly awaiting the selection and implementation of a package of evidence-based programs. Rather, they are usually overflowing and overwhelmed by their independent organizational mandates and full schedules (Dishion, 2011). They often have competing agendas due to a lack of integration among individual programs and across multiple systems (e.g., education, law enforcement, juvenile justice, mental health and human services). An unintentional fragmented approach to the prevention of problem behaviors seems to underlie this issue (Saul et al., 2008). For example, in many communities there are individual programs and organizations focused on drug prevention, violence prevention, pregnancy prevention, school dropout prevention, truancy prevention, and positive youth development. Consequently, communities and schools are left with a long list of what they need to accomplish but no map concerning how to integrate these approaches into a realistic and effective overall strategy.

This suggests a third systemic barrier to an evidence-based comprehensive approach—communities and schools often struggle to understand how a package of evidence-based programs can fit together to create a strategic, sustainable, evidence-based comprehensive approach. They are challenged with: collecting and using data to make decisions about program selection and impact; achieving consensus on the prioritized problems and the solutions; how to implement the programs with fidelity; how to create a hospitable environment for evidence-based programs to survive; and, when this approach involves multiple sectors and agencies (e.g., community and school), who has the authority and responsibility for ensuring its success (Mihalic & Irwin, 2003; Saul et al., 2008).

### Considerations for Matching Comprehensive Packages of Evidence-Based Youth Violence Prevention and Intervention Programs With Community Needs

Fortunately, the emerging field of implementation science is helping researchers and communities to understand what is needed to effectively implement and bring comprehensive packages of evidence-based interventions to scale (Aarons, Hurlburt, & Horwitz, 2011; Damschroder et al., 2009). Effective implementation is now recognized as an active process that can be done intentionally, studied in practice, and supported by funders and governments. Implementation science provides a critical roadmap to guide communities in the adoption, effective and efficient implementation, and sustainability of evidence-based programs (Kelly & Perkins, 2012).

Program selection and adoption is a foundational stage in this process. Careful, purposeful work to match youth violence prevention and intervention programs with community needs establishes a foundation for successful programs (Hawkins, 1999). The selection and adoption of evidence-based programs involve consideration of a variety of factors, including the characteristics of both the program and the community where it will be implemented, taking into account aspects such as the cultural and developmental relevance of the program, risk and protective factors associated with the target community, and the capacity or readiness for the community to support the program (Sullivan et al., in press).

Assessing community capacity or readiness takes into account all of the factors specified above and considers the broader social, economic, cultural, political, and policy contexts that may support or inhibit the success of a community in implementing a comprehensive approach to violence prevention and intervention. The degree of readiness or capacity within organizations (e.g., schools and community agencies) to effectively deliver prevention programs proves critical to implementation success. This includes both (a) *innovation*-*specific capacity*, or the fit between prevention programs and organizations’ day to day operations, priorities, and goals, and organizational buy-in as determined by the allocation of time, staff, and resources needed for effective program implementation (Flaspohler, Duffy, Wandersman, Stillman, & Maras, 2008); and (b) *general organizational capacity*, or the extent to which the organization’s infrastructure, climate and leadership fit with and support the prevention program (Flaspohler et al., 2008).

All organizations exist within a shifting ecology of social, economic, cultural, political, and policy environments that disparately and simultaneously enable and impede implementation and program operation efforts at the individual, community, state, and federal levels. Ideally, an enabling context exists that actively aligns federal and state efforts to support local comprehensive prevention initiatives. Some states (e.g., Pennsylvania and Washington) have built innovative state level prevention support systems to facilitate the adoption, implementation and sustainability of evidence-based programs by providing funding and technical assistance to build local capacity and research demonstrating outcomes and cost savings (Rhoades, Bumbarger, & Moore, 2012; Washington State Institute of Public Policy, 2014). These examples suggest that careful attention needs to be paid to creating readiness in attitudes, skills, and infrastructure at all levels before putting evidence-based programs into place.

Community capacity building expert Tony Karbo (2014) identifies key approaches to capacity building across multiple societal levels that can be applied to the effective implementation of evidence-based program packages that we describe here. He states that “All capacity-building activities must be anchored on a set of principles that will ensure and sustain trust and cooperation between those bringing in capacity programs and the intended beneficiaries” (Karbo, 2014, p. 21). Local communities are significant actors in preventing youth violence. However, creating an enabling context for violence prevention and intervention programs to thrive also involves coordination and alignment of capacity building interventions across organizational, community, state, and national efforts (Bursik & Grasmick, 1993). Achieving this alignment is a continuous long-term process and commitment that requires outside partners to focus on what communities truly need, and to ensure participation, inclusivity, and transparency in the process.

Given the intricacies of building community capacity for readiness, it is no surprise that studies of the adoption of evidence-based prevention programs in organizational contexts (e.g., schools and community agencies) suggest that the process is complex, organic, and messy (Greenhalgh, Macfarlane, Bate, & Kyriakidou, 2004). The complexity of this process is magnified when selecting and implementing comprehensive evidence-based program packages. This is an adaptive challenge, which by nature is complex, since the answer is not known. Even were it known, no single entity has the resources or authority to bring about the necessary change. In these cases, reaching an effective solution requires learning by all the stakeholders involved in solving the problem. Often these stakeholders are challenged with changing their own individual and organizational policies, programs and practices in order to create truly effective solutions (Kania & Kramer, 2011). Next we describe how researchers and communities worked together to overcome some of these adaptive challenges to develop comprehensive evidence-based program packages to prevent youth violence.

## Illustrative Examples From the Youth Violence Prevention Centers (YVPCs)

This section provides an overview and illustrative case examples of the main factors that were addressed in the adaptive challenge of matching an evidence-based comprehensive youth violence prevention approach with community needs across six CDC-funded YVPCs. At each YVPC site, the process of selecting prevention and intervention programs represented a partnership between researchers and community members that addressed the following factors: (1) community capacity, (2) researcher and community roles in selecting programs, (3) using data in decision-making related to program selection, and (4) reach, resources, and dosage along with the consideration of the synergy between the prevention programs and their additive contributions in addressing youth violence within each community.

Six YVPCs went through elaborate partnership processes in identifying and selecting programs for their comprehensive packages to reduce youth violence. Table 1 displays summary information on each of the YVPCs. These Centers are located in universities in disparate areas across the United States: Chicago, IL, Ann Arbor, MI, Richmond, VA, Boulder, CO, Baltimore, MD, and Chapel Hill, NC. Community partners were located in the same city (Chicago, Baltimore, Richmond), a nearby area (Flint, MI) or an area some distance from the university centers (Montbello community in Denver, CO; Robeson County, NC). The target communities were diverse in demographics; five out of six partnered with inner city neighborhoods in large metropolitan areas (Chicago, Baltimore, Denver) and smaller cities (Flint, MI, Richmond, VA). The North Carolina Center partnered with a rural county. All of the partner communities were coping with high levels of poverty, unemployment, and crime. Minority residents were strongly represented by large proportions of African Americans, Latinos, and American Indians (in the rural NC county).

**Table 1:** YVPC comprehensive program package selection summary information

	Chicago Center for Youth Violence Prevention	University of Michigan Youth Violence Prevention Center	Virginia Commonwealth University Clark-Hill Institute for Positive Youth Development	University of Colorado Boulder (CU-Boulder) Youth Violence Prevention Center	Johns Hopkins Center for the Prevention of Youth Violence	UNC-Chapel Hill: North Carolina Academic Center for Excellence in Youth Violence Prevention
Target community	Humboldt Park neighborhood in west Chicago, Illinois *N* = 37,000	Durant-Tuuri-Mott neighborhood in Flint, Michigan *N* = 9355	Three communities in Richmond, Virginia defined by middle school attendance zones *N* = 43,130	Montbello community in Denver, Colorado *N* = 30,000	Lower Park Heights community in Baltimore *N* = 12,000	Robeson County, NC. Near border of North and South Carolina *N* = 124,000
Key demographics	Urban. High poverty and crime Predominately African American and Latino	Urban. High poverty and crime. Predominately African American	Urban. High poverty and crime. Predominately African American and Latino	Urban. High poverty and crime. Predominately African American and Latino	Urban. High poverty and crime. Predominately African American	Rural. High poverty and crime. Large Native American population
Community capacity/readiness for implementation	Capacity moderate; 1 program was already occurring; community connections in schools; capacity for 1 program needed to be developed	Capacity moderate to high; 3 out of 6 programs already had evidence, community connections	Capacity low to moderate; none of the programs were occurring; University had a long history of working with schools; few community programs	Capacity low to moderate; 2 non-profit organizations and Mayor’s Office strong initial partners	Capacity moderate; University had a long history of working with the community on violence prevention	Capacity low; none of the programs selected were already occurring
Researcher and community role in selecting programs	School and community partners worked with researchers to develop a youth violence prevention plan that expanded existing programs (Cease Fire), addressed the need for family interventions, and to create a package of programs that included universal prevention programs and selective programs targeting youth at high risk	Researchers presented a draft program matrix and plan to the community for feedback. The research team has a strong history with the community and the selected. Final decision on program selection was made by the research team but included community input	Programs were selected in collaboration with the community based upon a needs assessment that included a review of existing programs and gaps within the school system. Evidence demonstrating program effectiveness was also a consideration in selection	The research team provided training and support to use the Communities That Care model; Community partners determined the top risk and protective factors, criteria for program selection, and selected programs based on their criteria from the Blueprints for Healthy Youth Development evidence-based program list	Community partners approached researchers about using Safe Streets to address violence; also the community emphasized need for jobs training to be included in their program package. At the school level, a gap in selected and indicated services was identified. Researchers met with school administrators to identify evidence-based programs that would fit well	Researchers created a menu of evidenced-based programs for community to choose from. Community gave program recommendations but some did not fit with research demand Community stakeholders described what had and had not worked in the past. Negotiated specific program components that were important to the community (e.g., Teen Court)
Data used in decision-making	Crime data Census data Evidence from program evaluation	Crime data. Census data. Community Survey. Evidence from program evaluation	Surveillance data. Census data. School and community youth violence data. Community needs assessment	Crime data. Community and school surveys	Crime data. School data	Crime data. Kids Count data from Annie. E. Casey Foundation School Success Profile-Plus data
Comprehensive package components	CeaseFire (Community) CeaseFire High School (School/Community) SAFE children (1^st^ Grade) (School/Family) GREAT Families (6^th^ grade) (School/Family)	Youth Empowerment Solutions (Individual) Fathers and Sons (Relationship) Clean & Green/Adopt- a-Lot (Community) ED Brief Intervention Mentoring Community Mobilization (Community Policing), crime prevention strategies	Olweus Bullying Prevention Program (School) After school youth leadership program (YES: School) Staying Connected with your Teen (Family) Parenting Wisely (Spanish: Family)	PATHS (Elem. School) Positive Family Support (Middle School/Family) Strengthening Families 10–14 (Family/Community) Safe2Tell (School/Community Positive Recognition Campaign (School/Community)	Safe Streets (Community) Positive Behavior Interventions and Supports (School) Middle school Coping Power (School)	School Success Profile assessment (School) Positive Action (Middle School) Teen Court (Community) Parenting Wisely (Family)
Selection barriers	No barriers in selection. Some programs had already started in the community (Cease Fire) and others had been successfully implemented previously in Chicago Public Schools (GREAT Families)	No barriers in selection. Several programs were developed in target community. Had to modify community policing because of low capacity in police department	A barrier was the lack of evidence-based programs available to choose from that were designed to meet the specific needs of our target population. Original plan to include community components was hampered by lack of infrastructure within the community	Initially building trust between researchers and community; community volunteers have limited time	High turnover of policy makers, school administrators, and service providers Difficult to maintain consistent supports when kids move to different neighborhoods that don’t have the programs	Distance to target community Strong insider/outsider mentality in community Competing demands, lack of support, in schools

*N* = the total population size for the target community’s geographic area

### Community Capacity

At the beginning of the funding period, community capacity varied across the YVPCs and was an important consideration in the development of comprehensive evidence-based packages. One YVPC partnered with a moderate to high capacity community; two YVPCs worked with communities with moderate levels of capacity; two YVPCs target communities were low to moderate; and one YVPC collaborated with a low capacity community. The level of community capacity influenced partnership development and the role of the academic partner.

In moderate to high capacity communities (i.e., partners in Chicago, Flint, and Baltimore), intervention programs were already present and may have been functioning for years. In Chicago, the Cease Fire program had functioned for a decade in the city. A community plan for youth violence prevention was already developed, and school leaders had a history of partnering with the University of Chicago. Similarly, University of Michigan researchers had longstanding ties with their community partners and had evidence supporting three out of six interventions that would be included in their comprehensive approach. Even though existing interventions had not been previously integrated into a comprehensive package, the process of packaging evidence-based programs was much more straightforward in moderate to high capacity communities with experienced partners. In these contexts, packaging largely meant bringing together existing resources into a coherent new system. Partners were motivated and had already initiated planning. The YVPCs added new resources to support, organize, and evaluate current efforts. They also added new programs that would complement the existing ones, creating a comprehensive approach. The moderate to high community capacity greatly facilitated the speed and efficiency with which the comprehensive approach could be designed and implemented.

In contrast, partnerships in lower capacity communities struggled more in the beginning because of the dearth of existing resources, disorganization, and lack of pre-planning. Interventions were not already present in the community that could be easily packaged and expanded to serve a greater number of community members. Schools were investing their energy elsewhere and, in some cases, were wary of outsiders asking to conduct research. There was an additional need to form trust with partners who did not have previous relationships with the YVPC universities. Forming trustworthy relationships was necessary, but slowed down the planning process. In Denver, researchers following the Communities That Care strategic planning process spent 18 months building capacity and creating readiness before program implementation could begin. In some cases, such as Richmond, variability in community capacity was found with high levels of capacity and long-standing partnerships in some areas (i.e., with the city school district) that facilitated selection and implementation of evidence-based prevention efforts in schools but with lower levels of community capacity in other areas (e.g., the infrastructure necessary to support community-based programs).

### Researcher/Community Roles in Selecting Programs

In the spirit of academic-community partnership, YVPC researchers always worked in collaboration with community partners. Collaborative roles, however, are not always equal; in some cases the community partners led and in other situations, researchers led the process. The overarching goal for the partnership was to sift through evidence of what works, identify and align programs with community needs, and ultimately create a coherent, systemic framework for the new initiative. For example, researchers from Johns Hopkins Center for the Prevention of Youth Violence supported programs the community requested. A strong community non-profit had convened community meetings, resulting in a request for Safe Streets/Cure Violence with a focus on jobs to be included in the comprehensive package. Schools were already implementing Positive Behavioral Interventions and Supports (PBIS); however, a gap in selected and indicated services was identified. Researchers met with school administrators to identify programs that would fit well. This is an example of a moderate capacity community prioritizing programs and leading the process. University of Michigan researchers similarly worked with partners who chose existing programs that met the evidence-based criteria required by CDC. Researchers presented the overall matrix with supplementary programs added. Due to the long history of collaboration, the novelty offered by the researcher partners lay in bringing the existing programs into a comprehensive initiative.

Low capacity communities needed more leadership from research partners to guide them through the selection of evidence-based programs. While still collaborative, researchers were more directive in these circumstances. For example, the University of Colorado Boulder (CU-Boulder) team provided training and support to use the Communities That Care model. They guided the process by (a) working in partnership with the community to create and train a community and key leader advisory board to oversee the initiative and (b) providing data to the boards about community risk factors and evidence-based programs using the Blueprints for Healthy Youth Development Program list as the menu (Blueprints for Healthy Youth Development, 2015a, b). However, the community developed a set of criteria and made the final decision about the selection of programs that fit their local needs and context. North Carolina researchers similarly led community partners through an examination of needs assessments collected from middle school students. They presented a menu of evidence-based program options to community stakeholders. The resulting package after extensive discussions included a well-known model program, a family intervention that had substantial evidence of effectiveness, and a teen court program that community members had previously implemented and wanted to improve.

### Using Data in Decision-Making Related to Program Selection

All of the YVPCs used some type of data to inform program selection and placement. Use of crime and census data was common across sites for identifying community hot spots in need of intervention. A variety of data sources (e.g., school and community surveys, child and family well-being data) were used to determine malleable risk and protective factors for youth violence at various socio-ecological levels within each community (e.g., individual, family, peer, school, and neighborhood). Data that assessed problem behaviors, as well as risk and protective factors, strengthened each site’s ability to ensure that the selection of evidence-based prevention programs fit community needs (Catalano et al., 2012). Prioritized risk and protective factors were matched with potential evidence-based programs that addressed these factors based on level of need (i.e., universal vs. high-risk populations) and developmental, cultural, and contextual relevance.

The types of data used and the processes for using data in program selection varied across the YVPCs. Needs assessments were used by YVPCs that needed extra structure, organization, and new information to inform their program selection process. Virginia Commonwealth University researchers had a long history of working with the Richmond schools on program implementation, but found that infrastructure was sparser within broader community settings. They completed a community needs assessment in Richmond that suggested that youth, parents, and service providers lacked knowledge of available youth programs, supports and resources. This new information was utilized in crafting their youth violence prevention initiative. Additionally, researchers working with their targeted communities for the first time tended to have less specific information, increasing the need to conduct needs assessments and to use community-level models of decision-making that begin with needs and gaps analyses. For example, Denver used the Communities That Care prevention system (Fagan, Arthur, Hanson, Briney, & Hawkins, 2011; Hawkins, 1999) to identify key risk and protective factors and develop strong relationships with community partners. Researchers from CU-Boulder collected baseline data using community household surveys (youth and parent) and a school survey and led partners from the Denver neighborhood through a process to prioritize the top three to five risk and protective factors in the schools and in the community. In North Carolina an extensive survey was conducted, randomly sampling 40 % of middle school students in the target community and comparing their responses to a full census of middle school students in the comparison county (total sample exceeded 4500 adolescents). These needs assessments allowed the partnership teams to balance evidence-based programming with specific community needs.

Several sites had worked within their target communities for a number of years and had strong, ongoing relationships with community partners (Chicago, Flint, Michigan, and Baltimore). Many of these sites had existing data (e.g., qualitative studies of risk and protective factors, surveillance data, needs assessments) and prior input from community partners that informed their selection of evidence-based programs. Community conversations had already identified key needs and there were histories of evidence-based programs already targeting key risk factors. These higher capacity sites could thus skip the identification of needs and expedite program selection based on existing activities. They concentrated on bringing extant programs together into a comprehensive initiative and making sure there was a good fit among intervention components. However, all sites are using assessment data to measure the impact over time of the comprehensive program packages on youth violence and other problem behaviors (Farrell, Henry, Bradshaw, & Reischl, 2016).

Along with needs assessment data unique to the target communities, partnership teams also focused on identifying programs with past evidence of program effectiveness. This information was drawn from national archives, such as Blueprints for Healthy Youth Development or SAMHSA’s National Registry of Evidence-Based Programs and Practices (NREPP; Blueprints for Healthy Youth Development, 2015a, b; SAMHSA’s NREPP, 2015). If community members asked for a favored program to be included in the initiative, it was incumbent upon researchers to examine the effectiveness data on that program and to share these assessments with community partners. In higher capacity sites, the researchers and community partners may have generated evidence of program effectiveness from past activities. There was less reliance on national program archives. This allowed the partners to efficiently move through program selection and launch the initiative faster. They were also able to concentrate attention on the fit among different program components.

### Reach, Resources, and Dosage

In addition to risk factor identification and program selection, YVPCs had to deal with reach, resources, and dosage in planning their youth violence prevention packages. The term reach refers to the number of people served by the package’s programs. YVPC teams had to balance how to make the greatest impact in their target communities with the available resources or funding and the planned dosage or intensity of the initiative. This calculation was different for each YVPC site. Sites working with inner city neighborhoods were highly concentrated in a relatively small area across several census tracts, police beats, or school catchment zones. Concentrating an intensive intervention program like Cease Fire or Safe Streets within a few neighborhood blocks maximized the program dosage in these neighborhoods. The catchment area often had two or three schools to work with. At the other extreme, the rural county in North Carolina was 925 square miles with 13 middle schools to serve. Having adequate program reach across such a large area impacts program dosage and uses a great deal of resources. Assuming that funding levels were fixed, important decisions needed to be made concerning how to make an impact that would significantly benefit the community, including which participants to target in order to accomplish this and at what intensity for program dosage. Readiness and capacity for implementation played greatly into these decisions across the sites. This balance between reach, resources, and dosage influenced the development of program packages and their implementation.

The six YVPCs also considered the potential synergistic and additive nature of prevention and intervention programs (Domitrovich et al., 2010). Within a comprehensive approach to youth violence prevention, multiple interventions were often needed within one context (e.g., family, school, or neighborhood) or across several contexts to effectively address a set of risk and protective factors related to youth violence (Nation et al., 2003). Using data driven approaches and theory to guide the selection process, the YVPCs selected a combination of prevention and intervention programs that had the broadest range and scope based on available resources to address the risk and protective factors for youth violence within a specific community (Domitrovich et al., 2010). This involved the prioritization of community needs and in some cases the ability to leverage or build upon existing programs and capacity in determining the final package of prevention programs.

Another synergistic effect of this approach is its potential for a broad impact on multiple problem behaviors and positive youth development. Since these programs address the underlying risk and protective factors that predict multiple problem behaviors, the programs selected are likely to also affect outcomes beyond youth violence (e.g., substance use, pregnancy prevention, school dropout, mental health). In fact, some of the evidence-based programs selected in the program packages support the acquisition of underlying master skills (e.g., social-emotional learning core competencies) considered necessary for successful human development (Elias et al., 1997; Jones & Bouffard, 2012; Osher et al., 2007). Recognition of this helped to unify partners across sectors (e.g., schools, law enforcement, mental health, juvenile justice) to support this comprehensive approach.

### Resulting Comprehensive Program Packages

Each YVPC worked through the processes articulated above (assessing community capacity, delineating researcher and partner roles, using data in decision-making, balancing reach, dosage and resources) to construct a comprehensive youth violence prevention initiative. The final programs included in each YVPC package are shown in Table 1. The packages were organized to target multiple ecological levels (e.g., individual, family, peers, school, and neighborhood) and universal and high-risk components. The final packages were a comprehensive mix of programs that balanced evidence of effectiveness that the researchers advocated for and programs requested by community partners tailored to meet each community’s specific needs.

## Discussion

### Barriers, Lessons Learned and Opportunities for Policy and Practice

As demonstrated in the examples discussed above, academic-community partnerships effectively supported the selection of comprehensive evidence-based program packages that were both grounded in research and responsive to individualized community needs.

Overall, the YVPCs are building a sustainable infrastructure for prevention of violence and problem behaviors at the community level–providing concrete examples for integrating community and practitioner perspectives with prevention science (Fagan, Hanson, Hawkins, & Arthur, 2009; Spoth & Greenberg, 2011). This front-end work of careful program selection also establishes an infrastructure for implementation with fidelity and for sustainability (Cooper, Bumbarger, & Moore, 2015). Although each of the six YVPCs varies in many ways, common barriers, lessons learned, and opportunities for policy and practice have emerged across the sites that demonstrate how to translate prevention science into community practice to develop and implement comprehensive packages of evidence-based youth violence prevention and intervention programs matched to community needs (Saul et al., 2008).

First, researchers played a critical role in providing data, resources, and technical assistance to help communities prioritize their prevention and intervention needs and to select a package of evidence-based programs that fit their specific context. Researchers have access to critical research knowledge (i.e., academic databases, archives, statistical interpretations) that is not easily accessible at the community level. Researchers can inform and provide support for data-driven decision making at this level. This includes providing surveillance data and support in how to understand and prioritize community youth violence prevention needs (Masho, Schoeny, Webster & Sigel, 2016). Additionally, at the community level, there is much confusion around the meaning of evidence-based programs and how to evaluate local programs. Community members want programs in their community that will produce desired results. Researchers can play a key role in supporting communities to accomplish what they want to achieve by providing technical assistance that builds community capacity to make good decisions in program selection. However, to truly be sustainable, the prioritization and ownership of the initiative must ultimately lie within the community. The researcher-community relationship worked best when there was a mutual understanding that the final decisions on evidence-based program selection would involve a partnership that promoted community knowledge and ended in the selection of evidence-based prevention and intervention programs that best met community needs. Understanding and responding to the local context and needs, while providing guidance on what research shows is effective, were common practices across all six sites.

Second, there is no quick way to select comprehensive packages of evidence-based programs. The sites that were able to select their program packages at a faster rate had been working with the community for approximately 10 years. These communities already had developed trusting relationships with their academic institution. In all six sites, trust has been built between the researchers and community and the relationship has moved from insider–outsider to a mutually beneficial partnership. Getting to this place required listening and honoring community needs, following through on promises, building capacity for evaluation at the community level, and finding ways to make things easier on already taxed systems.

Barriers to smooth program selection and implementation always arise, especially in fashioning complex program packages for large scale implementation. Some YVPCs had to eliminate or scale back program implementation ideas because of low capacity in police departments, limited time and competing demands for community partners, high turnover of service providers and policy makers, lack of support, or lack of evidence-based programs available to choose from that were designed to meet the specific needs of the target population. Not all the barriers were based in the community. In Year 3, funding levels were reduced for the YVPCs, necessitating a re-evaluation of how limited resources would be used in each site. Universities often work on different schedules compared to communities, making these institutions less nimble in responding to day-to-day turbulence. It can take months to get a new budget item approved by university administration and the funder. Each of these obstacles required creative problem solving and negotiation. These barriers were minimized in all the sites by choosing programs that were already started or very important to the community. Being a true partner with the community also means helping out with various community needs: supporting existing community events, and selecting community members for key roles in leading the youth violence prevention programs whenever possible.

Third, capacity building for prevention is a long-term process. As demonstrated across the YVPCs, the process of building capacity must be flexible in addressing the needs of the community by meeting them at their stage in the developmental process. Therefore, the national approach to supporting these efforts should not be inconsistent or fragmented. Staying the course for the long-term is critical for building trust, organizational learning, and effective implementation. Long-term academic-community partnerships can facilitate building linkages at the local, state and federal levels to align resources to support identified community needs. Ideally, strategic academic-community partnerships should last over the course of decades.

It is important to note that each community selected as part of this project demonstrated high rates of youth violence and was situated in a low-income area. Communities in these areas may face a myriad of stressors that contribute to high levels of youth violence. Some communities also had low levels of capacity for the prevention efforts, which necessitated considerable time spent in developing readiness prior to the implementation of these programs. Thus, the experiences described in initiating prevention programs for the selected communities may not generalize to communities with higher levels of socio-economic status.

## Conclusion

There continues to be a significant gap between what is known to be effective in preventing and addressing youth violence (e.g., a comprehensive evidence-based approach) and what programs and strategies are actually implemented. To achieve the public health impact that has been demonstrated to be possible in randomized trials, this gap must be narrowed. This paper provides concrete examples of six YVPCs across the nation actively closing the gap between science and the practice of prevention by selecting, implementing, and evaluating comprehensive packages of evidence-based programs. The development of model comprehensive systems to move these evidence-based program packages toward population health improvement is still in its infancy. However, the lessons learned across the six YVPCs provide suggestions and examples for researchers, policy makers, practitioners, and other community partners that can make this approach easier to apply in other communities.

Careful program selection grounded in research, but tailored to each community’s specific needs, is foundational to the success of a comprehensive evidence-based approach. Communities and schools often struggle to understand how a package of evidence-based programs can fit together to prevent youth violence and other problem behaviors. Researchers can play a critical role in providing data, resources, and technical assistance. However, these data become particularly meaningful when data are vetted and viewed through the lens of local community members since they know what programs will fit and flourish within their local context.

As this article suggests, the process of selecting comprehensive evidence-based program packages is complex, organic, and messy and challenges always occur. Although there is no one-size-fits-all approach, there are some key ingredients for successful collaborations. Trust between the researchers and the community is essential and requires a continuous long-term process that requires researchers to focus on what communities truly need and to ensure participation, inclusivity and transparency (Karbo, 2014). For the six YVPCs, this has resulted in a learning environment that is mutually beneficial to both the researchers and community partners. Researchers respond to the local context and needs, while providing guidance on what research shows to be effective to prevent violence. Communities learn how to use their local data to make decisions on program selection and implementation and receive much needed funding and infrastructure support.

There are no shortcuts to this work. Strategic academic-community partnerships to create and implement comprehensive evidence-based program packages to prevent youth violence take time and significant investment to build trust, to allow time to demonstrate community-level outcomes, and to ensure sustainability. While shifting to an evidence-based comprehensive approach requires considerable change and resources, it offers the greatest potential to prevent youth violence and collectively impact the successful development of children, families, and communities.
